# Combining High-Pressure NMR and Geometrical Sampling to Obtain a Full Topological Description of Protein Folding Landscapes: Application to the Folding of Two MAX Effectors from *Magnaporthe oryzae*

**DOI:** 10.3390/ijms23105461

**Published:** 2022-05-13

**Authors:** Cécile Dubois, Mounia Lahfa, Joana Pissarra, Karine de Guillen, Philippe Barthe, Thomas Kroj, Christian Roumestand, André Padilla

**Affiliations:** 1 Centre de Biologie Structurale, University of Montpellier, INSERM U1054, CNRS UMR 5048, 34000 Montpellier, France; ceciledubois98@gmail.com (C.D.); mounia.lahfa@cbs.cnrs.fr (M.L.); joana.pissarra@cbs.cnrs.fr (J.P.); karine.deguillen@cbs.cnrs.fr (K.d.G.); philippe.barthe@cbs.cnrs.fr (P.B.); andre.padilla@cbs.cnrs.fr (A.P.); 2 PHIM Plant Health Institute, University of Montpellier, INRAE, CIRAD, Institut Agro, IRD, 34000 Montpellier, France; thomas.kroj@inra.fr

**Keywords:** protein folding, NMR, high hydrostatic pressure, *Cyana3* calculations, MAX effectors

## Abstract

Despite advances in experimental and computational methods, the mechanisms by which an unstructured polypeptide chain regains its unique three-dimensional structure remains one of the main puzzling questions in biology. Single-molecule techniques, ultra-fast perturbation and detection approaches and improvement in all-atom and coarse-grained simulation methods have greatly deepened our understanding of protein folding and the effects of environmental factors on folding landscape. However, a major challenge remains the detailed characterization of the protein folding landscape. Here, we used high hydrostatic pressure 2D NMR spectroscopy to obtain high-resolution experimental structural information in a site-specific manner across the polypeptide sequence and along the folding reaction coordinate. We used this residue-specific information to constrain *Cyana3* calculations, in order to obtain a topological description of the entire folding landscape. This approach was used to describe the conformers populating the folding landscape of two small globular proteins, AVR-Pia and AVR-Pib, that belong to the structurally conserved but sequence-unrelated MAX effectors superfamily. Comparing the two folding landscapes, we found that, in spite of their divergent sequences, the folding pathway of these two proteins involves a similar, inescapable, folding intermediate, even if, statistically, the routes used are different.

## 1. Introduction

Contrary to the “classical view” of protein folding, that describes folding in terms of a defined sequence of states along the reaction coordinate axis, the “new view” of protein folding replaces this single-pathway model with trajectories on a rugged energy landscape [1,2,3,4]. Upon this energy landscape, the specific trajectory taken by a given molecule is determined by thermodynamic probabilities [2]. This statistical model predicts that multiple pathways between the folded and unfolded states must exist for all proteins [3]. Whether all trajectories between states remain possible at the single molecule level, the probability of a particular molecule taking a specific pathway depends on the starting conformation of the polypeptide chain, allowable thermal motion and relative height of energetic barriers.

Nevertheless, there is very little experimental evidence for the existence of such parallel folding pathways [5,6,7,8,9,10,11]; the most unequivocal experimental evidence was provided by the Ig-like domain I27 (also called I91) of the giant muscle protein Titin [10,11]. This is probably because of the lack of techniques allowing a full (or even simplified) description of the folding landscape. Indeed, the full description of a protein folding reaction requires understanding of how the environment of each atom—at least, of each residue—from each individual protein in solution evolves during the reaction. This is far beyond what was achieved with most of the protein folding studies, relying on the measurement of global physical and spectroscopic observables, such as fluorescence, circular dichroism, FTIR or SAXS. These techniques give access to ensemble averages and lack the desired spatial (structural) or temporal resolution. Single-molecule techniques using Förster resonance energy transfer (FRET) [12] or IR spectroscopy [13] have enough temporal resolution to describe the heterogeneity of the molecule ensemble, but lack high spatial resolution. In addition, with these techniques, the acquisition of site-specific information at multiple points of the polypeptide chain requires the separate preparation of protein variants modified at each residue (or pair of residues) of interest and a separate set of experiments for each.

Multidimensional NMR spectroscopy is one of the experimental techniques with the potential to contribute to a high-resolution, site-specific, time-resolved description of the protein folding reaction. This is essentially due to (i) the extreme sensitivity of NMR observables to the structural environment, and (ii) the fact that an abundance of site-specific probes can be studied simultaneously in a multidimensional NMR spectrum [14]. Even if this technique can be combined with the “classical” chemical or temperature perturbations, when combined with high-hydrostatic pressure perturbation it can yield unprecedented details on protein folding pathways [15,16,17,18,19,20]. Thus, the probability of contact between specific residues was readily measured from residue-specific denaturation curves obtained from NMR data, and used to constrain Go-model calculations, allowing the characterization of the structure and energetics of the folding landscape of different proteins and the identification of major folding intermediates [21,22]. Nevertheless, in these previous studies, the length of the MD calculations restricted the description of the folding landscape only at a given pressure, precluding the characterization of all the conformers populating the landscape along the full pressure axis (1–2500 bar). Here, we propose an alternative method that allows the full structural description of the conformers populating the folding landscape during the folding/unfolding reaction. Among other things, we replaced the Go-model simulations with *Cyana3* calculations [23], a popular software commonly used to model the 3D structure of protein from distance restraints derived from NMR data. Working on the dihedral angle space, *Cyana3* allows considerably faster calculations than the Go-model simulations, allowing the complete exploration of the folding landscape within reasonable computational times.

**Figure 1:**
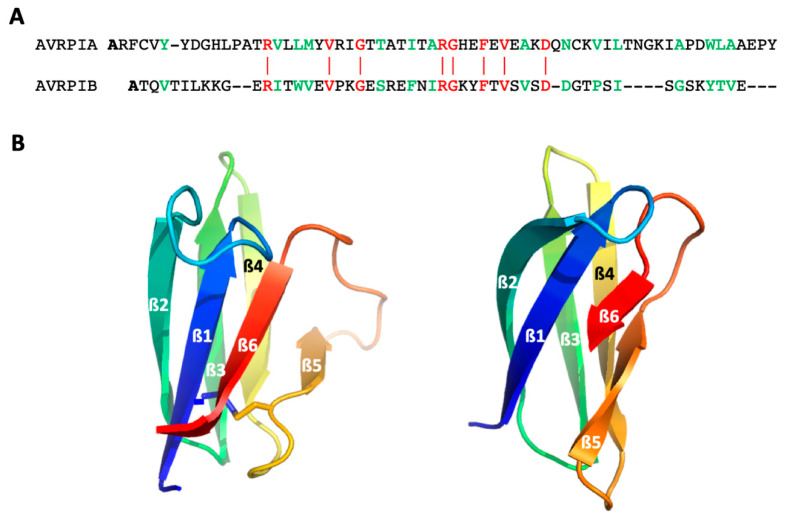
**The structure of AVR-Pia and AVR-Pib.** (**A**) sequence alignment (DALI [25]) of the two proteins. Residues colored in red correspond to identical residues in the two sequences. Sequence identity (≈12%) is further highlighted by the red vertical lines between the two sequences. Residues colored in green corresponds to homologous residues between the two sequences (≈35% sequence homology); (**B**) Cartoon representation of AVR-Pia (PDB: 6Q76 [26]) (left) and (**B**) AVR-Pib (PDB: 5Z1V [27]) (right) structures. The six strands are labeled ß1 to ß6, and the structures are colored from the N-ter in dark blue to the C-ter in red. The SS-bridge in AVR-Pia is shown by sticks.

We tested our approach on two small globular model proteins, AVR-Pia and AVR-Pib from the blast fungus *Magnaporthe oryzae*, belonging to the structurally conserved but sequence-unrelated MAX (Magnaporthe Avirulence and ToxB-like) effectors superfamily [24], which play an important role during biotrophic host colonization, and can, in some cases, circumvent host immunity. Despite their low sequence identity (≈12%), these two proteins display a similar 3D fold, characteristic of the MAX effector family: a sandwich of two three-stranded antiparallel ß-sheets, with an identical topology (Figure 1). Nevertheless, AVR-Pib lacks the usually well-conserved disulfide bond linking the two ß-sheets. In addition, to test our method the full description of the folding landscape for these two proteins may highlight more general questioning, such as whether proteins belonging to a same structural family have a similar folding pathway with similar folding intermediates? The answer to this question is expected to bring some clues about the way that a folding pathway is encoded by the primary sequence.

## 2. Results

### 2.1. High-Pressure Unfolding Monitored with NMR Spectroscopy

Crystal structures are available from the Protein Data Bank for AVR-Pia (PDB: 6Q76 [26]) and AVR-Pib (PDB: 5Z1V [27]) and were used as a template for displaying the results. In the case of AVR-Pia, two solution structures are also available (PDB: 2N37 [28], and 2MYW [24]), and the resonance assignment was deposited at the BMRB (n° 25460 for 2MYW). Nevertheless, the physical and chemical conditions used in these previous studies were different than those used in the present one, justifying the re-assignment of the amide resonances through guanidine titration experiments. The assigned HSQC for both proteins is given in the Supplementary Materials (Figure S1).

A series of 2D [^1^H,^15^N] HSQC spectra of ^15^N uniformly labelled proteins were recorded as a function of pressure in the conditions described in the Materials and Methods. As usual, we observed a decrease in the intensity of each native state peak as a function of pressure, with a concomitant increase in the corresponding unfolded state peaks, centered around 8.5 ppm in the proton dimension [20]. This strongly suggests a slow equilibrium between the chemical environment of each residue in the native and unfolded state on the NMR timescale, as well as a two-state transition between each native/unfolded cross-peak pairwise during the unfolding process. Since resonance assignments were available only for the native state structure of the two MAX effectors, the decrease in intensity with pressure for each corresponding cross-peaks was fitted to a two-state pressure-induced unfolding model, as described in the Materials and Methods (Equation (1); Supplementary Materials, Figures S2 and S3), yielding residue specific values for the apparent volume change ($\Delta V_{f}^{0}$) and apparent free energy ($\Delta G_{f}^{0}$) of folding. Accurate fitting was obtained for 50 residues over 61 non-proline residues for AVR-Pia and for 39 residues over 49 non-proline residues for AVR-Pib, giving a substantial number of local probes for the description of their folding pathways. They correspond to residues with which corresponding cross-peaks do not overlap, neither in the folded state of the protein nor in between the folded and unfolded states. Residue-specific $\Delta V_{f}^{0}$ and $\Delta G_{f}^{0}$ values measured for the two proteins are given in the Supplementary Materials (Figure S4). Average values of $\Delta G_{f}^{0}$ of −1769 ± 337 cal/mol and of −1885 ± 395 cal/mol were found for AVR-Pia and AVR-Pib, respectively. Note that these values do not inform about the relative stability of the two proteins, since the denaturation studies were conducted in different experimental conditions (4.5 M and 1.5 M GuHCl for AVR-Pia and AVR-Pib, respectively). On the other hand, the similar average values of $\Delta V_{f}^{0}$ found for the two proteins (49 ± 7 mL/mol and 44 ± 12 mL/mol for AVR-Pia and AVR-Pib, respectively) suggest similar global values for their internal voids inside the 3D structures, since this parameter exhibits a moderate dependency with the guanidine concentration [29].

**Figure 2:**
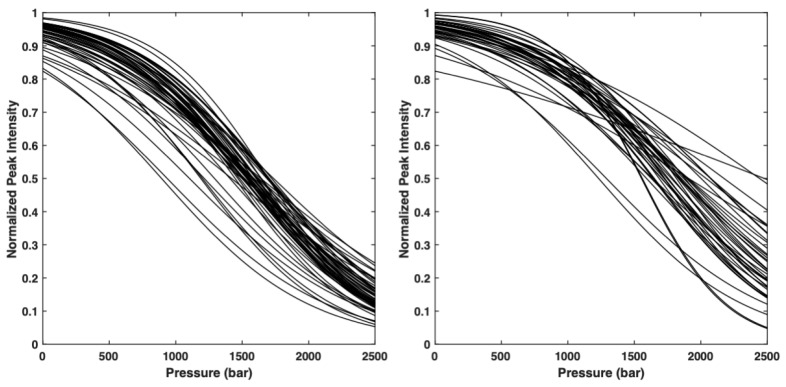
**Overlay of the normalized residue-specific denaturation curves** as obtained from the fit of the pressure-dependent sigmoidal decrease in the native cross-peak intensities in the HSQC spectra of AVR-Pia (**left**) and AVR-Pib (**right**) with Equation (1).

The normalized residue-specific denaturation curves are displayed in Figure 2. They give the probability *p_i_* for a given residue *i* to be in a native state at a given pressure. Thus, the probability of contact for any pair of residues, *p_i,j_*, at a given pressure, is defined as the geometric mean of the fractional probability of each of the two residues in the folded state at the same pressure $P\left(i,j\right)=\sqrt{p_{i\;}p_{j}}$ [22].

### 2.2. Determining Cut-Off Threshold for the Constraints Used for Topology Calculations

Instead of the Go-model simulations, used in a previous study to model the structure of conformers populated in the folding landscape at a given pressure [21,22], we chose to use *Cyana3* [23] to characterize the conformers populating the whole folding landscape for the two MAX effectors. Based on the torsion angle dynamics calculation, *Cyana3* presents the advantage to work directly on full-atom representations of the molecule, contrary to the Go-model that used simplified representations of the molecule, whilst maintaining a reasonable computational time. Moreover, when used without any experimental (distance) restraints, the force-field implemented in *Cyana3* is able to generate random conformers, the great majority of which have a correct geometry and satisfy the Ramachandran conditions (Supplementary Materials, Figure S5).

**Figure 3:**
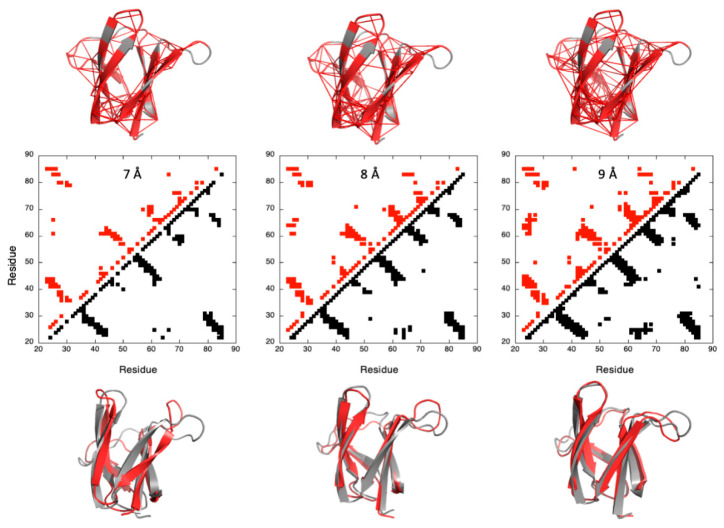
**Determining the cut-off threshold for the constraints used for *Cyana3* calculation of the structure of AVR-Pia**. Central panel: contact maps built from the X-Ray structure of AVR-Pia with cut-off threshold for the Cα–Cα distances of 7, 8 and 9 Å (from left to right), as indicated. Contacts below the diagonal correspond to residue where the distance to the corresponding Cα is lower than the threshold (black filled-squares). Above the diagonal, only the contacts for which fractional probability can be obtained have been reported (red filled-squares). In the upper panel, the corresponding Cα–Cα distances (110, 155 and 203 distances for cut-off thresholds of 7, 8 and 9 Å, respectively) have been reported on the X-ray structure. Residues involved in these contacts are also colored in red. The lower panel presents the results of the *Cyana3* calculations using the corresponding Cα–Cα distances as upper bound limit restraints. The best models (the closest to the X-ray structure) (red cartoons) are superimposed with the X-ray structure of AVR-Pia (grey cartoons): backbone heavy atoms <r.m.s.d.> of 4.79, 2.77 and 2.85 Å have been measured between the X-ray structures and the models obtained with cut-off threshold of 7, 8 and 9 Å, respectively (from left to right). A similar figure is available for AVR-Pib in the Supplementary Materials (Figure S6).

Thus, a first step in our analysis was to prove that a correct fold of the two MAX effectors could be obtained from *Cyana3* calculations, with a limited number of constraints that remained to be determined. To this aim, we built contact maps from the X-ray structures of AVR-Pia and AVR-Pib, using a different cut-off threshold for the inter-residue Cα–Cα distances. The contacts for which probabilities can be calculated from the denaturation curves were then used for the *Cyana3* calculations, using the corresponding Cα–Cα distances (+0.1 Å) as upper bound limits. Using a trial-and-assay approach (Figure 3), we selected a cut-off threshold of 9 Å, yielding 203 and 115 restraints for AVR-Pia and AVR-Pib, respectively, that were used for *Cyana3* calculations [30].

Average r.m.s.d values of 2.85 Å and 5.89 Å were measured between the obtained models and the X-ray structures of AVR-Pia and AVR-Pib, respectively (Supplementary Materials, Table S1). When introducing φ, ψ dihedral (±10°) restraints measured from the X-ray structures in the *Cyana3* calculations, these average r.m.s.d. values drop to 2.18 and 1.73 Å for AVR-Pia and AVR-Pib (Supplementary Materials, Table S1), respectively, yielding a significant improvement especially in the case of AVR-Pib, where less distance constraints were available. Moreover, reasonable spreading (average r.m.s.d.) within the conformer populations was observed. These dihedral restraints were then used in the following to improve the convergence of the calculations (see Supplementary Materials). Note that this increase in the convergence of the *Cyana3* calculations is only due to an improvement of the local geometry of the secondary structure elements (β-strands) present in the 3D structure of the MAX effectors. Introducing dihedral restraints can in no way have an influence on the spatial arrangement of the different β-strands during the folding process.

### 2.3. Simulations of Pressure Unfolding

We applied constrained *Cyana3* calculations to characterize the pressure-dependent unfolding process during which a protein undergoes transitions from a native compact structure to extended configurations. An ensemble of constraints’ lists, generated through filtering the native constraints by the probability *p_ij_* values obtained from the normalized denaturation curves at each pressure, was used to compute the conformer ensembles by the torsion angle dynamics software *Cyana3* (see Supplementary Materials). The total conformer ensemble sizes were similar for the two MAX effectors, with 23,108 conformers and 25,376 conformers for AVR-Pia and AVR-Pib, respectively. For each protein, the total set of conformers obtained for all the pressure ranges was sorted according to the fraction of native constraints (Q).

#### 2.3.1. Conformational Landscapes of AVR-Pia and AVR-Pib

**Figure 4:**
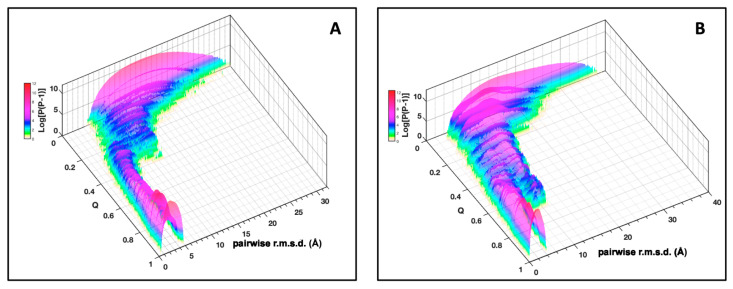
**Distribution of pairwise r.m.s.d.** for AVR-Pia (**A**) and AVR-Pib (**B**). The pairwise r.m.s.d. for a population of conformers (P) at each value of Q is given along the logarithmic vertical axis (Log[P(P − 1)]). These r.m.s.d. were binned at 0.125 Å.

The plot of the pairwise r.m.s.d. distribution of the AVR-Pia conformers (all vs. all) at increasing native fraction Q (Figure 4A) shows how the topological sampling converges towards the Fighu native structure. From low Q to high Q, we observe a redistribution of the pairwise r.m.s.d. landscape, starting from a broad distribution centered at approx. 15 Å at low Q, followed by a progressive narrowing of the distribution and a decrease in the pairwise r.m.s.d. center value at ~12 Å for Q = 0.2 and, concomitantly, the appearance of a novel population of pairwise r.m.s.d. at approximately 5 Å. This population becomes the majority, at Q above 0.3 and finally the only observed population at high Q values with a distribution centered at 3 Å. These general trends are also present for AVR-Pib (Figure 4B), although differences are observed in the pairwise r.m.s.d. distributions and the degree of convergence along the Q dimension.

However, the global analysis by pairwise r.m.s.d. distributions, while showing detailed differences between the two proteins, is difficult to interpret at the level of individual conformers. Moreover, it must be indicated that the generated conformational space is not uniformly sampled. At low and high values of Q, the conformational space is oversampled compared to the intermediate values of Q. We solved that situation by calculating a large number of conformers (>20,000), ensuring a sufficient distribution and coverage even at intermediate values of Q.

#### 2.3.2. Spreading of Conformations and Clustering

**Figure 5:**
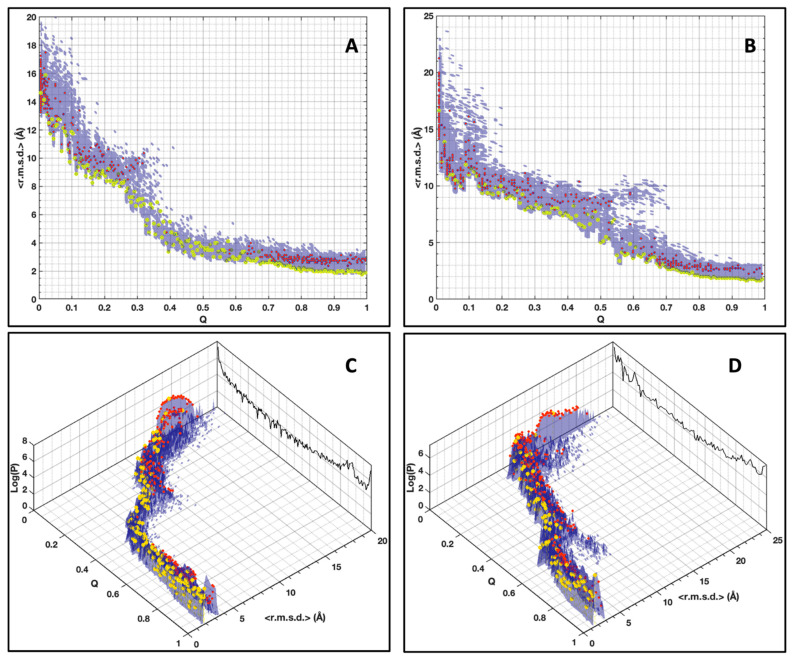
**Distribution of the conformers’ population** (Log scale) in relation to their <r.m.s.d.> (bin size 0.125 Å) and their native constraints fraction (Q) for AVR-Pia (**A**,**C**) and AVR-Pib (**B**,**D**). Cluster centroids are indicated by the red dots. The centroids for the most populated clusters are indicated by the yellow dots. The total conformer populations (Log scale) vs. Q are projected on the back-planes (black color) in panels (**C**,**D**).

The conformational landscape spreading is shown by computing for each conformer a mean <r.m.s.d.> averaged over the whole population of conformers at a given Q value (Figure 5). These plots highlight more details in the convergence profile along the Q dimension and allow mapping of the exact position of all the conformers in the landscape.

For both proteins, the <r.m.s.d.> are centered on ~15 Å at low Q, and progressively decrease to 10 Å at Q = 0.2, while details of the spreading of conformer populations are not identical. For Q in the range of 0.2–0.4 the <r.m.s.d.> rapidly decreases to 4 Å for AVR-Pia, while it is smoother for AVR-Pib and stays at 8 Å for Q = 0.4. Subsequently, in the last Q segment, the final convergence starts at an initial value for AVR-Pia that is smaller than in the AVR-Pib landscape. For AVR-Pib, we observe also a secondary branch starting at Q = 0.5 deviating from the major conformer profile that corresponds to inversions of the conformer topology relative to the native topology (Supplementary Materials, Figure S7C), where the ß3 and ß4 strands cross each other. By qualitatively comparing the spreading of the landscapes, we draw the conclusion that conformer diversity, seen by its larger distribution and heterogeneity, is greater in the case of AVR-Pib than for AVR-Pia.

A clustering approach was used to pool similar conformers into families giving, in fine, a schematic view of the conformer populations. To avoid over-simplification, the clustering parameters of the MaxCluster software were fine-tuned (see Supplementary Materials). The Nearest Neighbor clustering [31] success over all conformers was 86% and 85% for AVR-Pia and AVR-Pib, respectively. The cluster centroids were superimposed on the <r.m.s.d.> landscapes in Figure 5. One must be careful that the <r.m.s.d.> of a cluster centroid (having the smallest r.m.s.d. to all other conformers in the cluster) indicates the position in the landscape of the most representative conformer of all conformers in the cluster at a given Q value, without direct information about the spreading of the conformers in this particular cluster. Examples of clusters at Q = 0.5 are given in the Supplementary Materials (Figure S7) at Q = 0.5 for AVR-Pia (one cluster) and AVR-Pib (two clusters). The clustering approach has a remarkable feature that is that the most populated clusters have the tendency to be the ones having the smallest centroid <r.m.s.d.>, with a few exceptions located mostly at low Q values (below ≈0.05 and ≈0.08 for AVR-Pia and AVR-Pib, respectively).

As shown in Figure 5B at Q ≥ 0.9, we observe heterogeneity with more than one of the conformer clustered populations. The Cα–Cα distance constraints supplemented by the φ, ψ dihedral restraints at this final range of Q do not allow full convergence to the native structure. This is especially the case for AVR-Pia where 13% of the conformers display average r.m.s.d. of 2.17 Å, computed between the MP cluster centroid and the other cluster centroids at Q = 0.99. For AVR-Pib, only 0.5% of the final stage conformers (Q ≥ 0.9) do not belong to the main cluster.

#### 2.3.3. Energy Profiles

**Figure 6:**
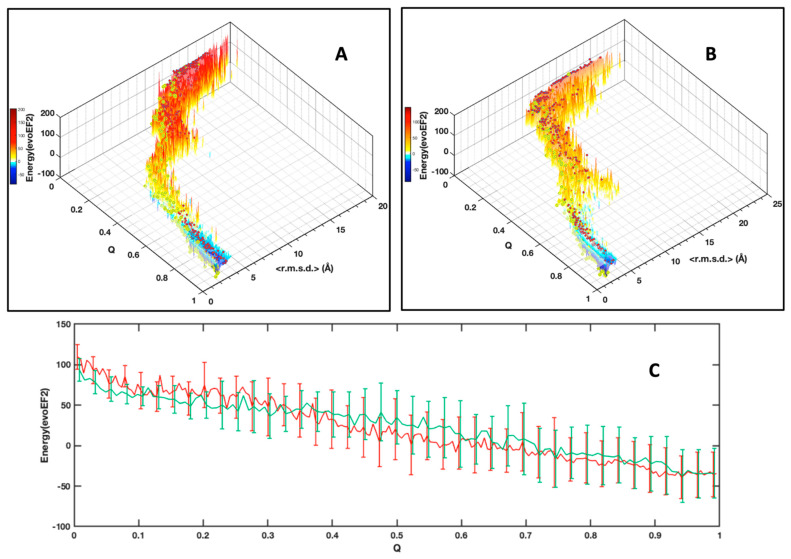
**Distribution of the conformers free energy calculated using the evoEF2 software** according to their <r.m.s.d.> (bin size 0.125 Å) and their native constraints fraction (Q) for AVR-Pia (**A**) and AVR-Pib (**B**). As in Figure 5, cluster centroids are indicated by the red dots. The centroids for the most populated (MP) clusters are indicated by the yellow dots; (**C**) Plot of the average free energy of the most populated clusters, for AVR-Pia (red) and AVR-Pib (green). The standard deviations (shown at 0.5 σ) are indicated by vertical bars. For clarity, the standard deviation bars were regularly spaced. The gain in free energy upon folding is ΔΔG_fold_ = −47 kcal/mol and −41 kcal/mol for AVR-Pia and AVR-Pib, respectively.

We calculated the free energy (in EvoEF2 energy units [32], see Supplementary Materials) of all the conformers and reported the values in Figure 6. At low Q, the conformers fluctuate with an average high energy, whereas the increase in Q is accompanied with a slow decrease in the average energy level, which becomes negative at high Q values, as can be seen for the most populated clusters (Figure 6C). This general trend is related to the increase in better Van der Waals packing and H-bonds energy terms through ‘favorable’ interactions being formed in the conformers when converging to the native structure (Supplementary Materials, Figure S8). We also observe elevated energy values spreading, as shown by the standard deviations reported in Figure 6C that are related to the sensitivity of the evoEF2 energy force field to differences of the conformer atom positions, even within the MP cluster.

#### 2.3.4. Inter-Cluster Trajectories Analysis

In the following, a trajectory along the Q dimension refers to an ensemble of links between cluster centroids. Despite the level of simplification obtained by clustering, the number of possible inter-cluster trajectories along the Q dimension still prevents the exploration of all possible inter-cluster trajectories. In any case, given the discontinuous nature of the sampling of the conformational space, we would not have access to a true trajectory as could be provided by molecular dynamics. We decided instead to explore the trajectory joining the most populated (MP) clusters, referred as the MP-trajectory. This trajectory gives a simplified vision of the topological folding scenario that allows focusing on the most relevant conformer features.

**Figure 7:**
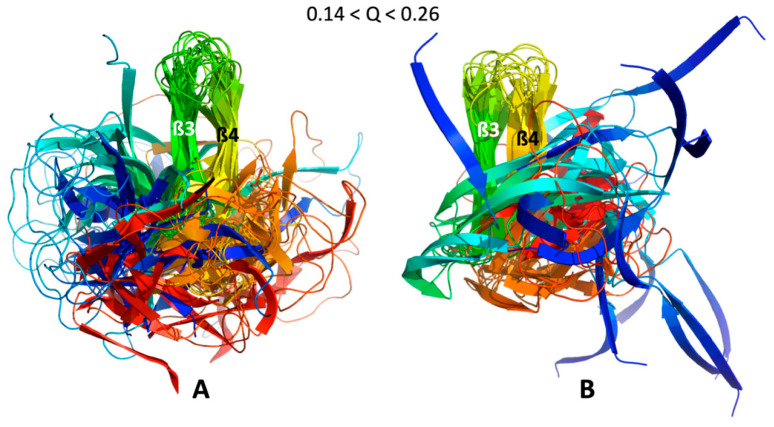
**ß3ß4 folding intermediates.** Cartoon Superimposition of the conformer centroids in most populated clusters selected to build the videos given in the Supplementary Materials (Videos S1 and S2), for (**A**) AVR-Pia and (**B**) AVR-Pib. Conformers centroids within the range 0.14 < Q < 0.26 are in rainbow colors from N-ter (blue) to C-ter (red) and the strands ß3 (green) and ß4 (yellow) are labelled.

Video frames build with MP-trajectories for AVR-Pia and AVR-Pib are given in the Supplementary Materials (Videos S1 and S2). Similar conformer intermediates could be identified in both of the video frames, as illustrated in Figure 7.

These folding intermediates involved the packing of two strands in the ß3ß4 sub-structure for both AVR-Pia and AVR-Pib, that we called the ß3ß4 folding intermediate. While the relative position of the other strands is more diverse for AVR-Pib, this is far from the case for AVR-Pia, where the ß1, ß2, ß5 and ß6 strands have the tendency to aggregate around the ß3ß4 aligned strands. Moreover, the observation of any folding intermediate needs to be statistically strengthened.

### 2.4. Statistical Relevance of the Simulations

The pressure data coverage (the percentage of residues for which a residue-specific denaturation curve was obtained) is 9% lower in the case of AVR-Pib than for AVR-Pia. The average restraint density (number of restraints per residue) is lower in the case of AVR-Pib than for AVR-Pia, with an average drop of two restraints per residue. Moreover, the restraint density is not homogeneous along the sequence for the two proteins (Supplementary Materials, Figure S9), preventing safe conclusions to be drawn from a direct analysis of the conformational landscapes. More generally, what we must question is the statistical significance of the generated conformational landscapes.

#### 2.4.1. Statistical Analysis of Clusters by Fractional Contact Maps

We make the hypothesis that the conformational landscape could possess biased distributions of conformers that are pressure-dependent. Accordingly, the underlying hypothesis is that hydrostatic pressure would provoke biases of the conformer population distributions by, for example, favoring some conformations compared to the conformations generated by random sampling. In that sense, the null hypothesis should be that hydrostatic pressure does not favor conformations (null bias) and that the analysis of the experimental data would not be significantly statistically different than a random conformational sampling, mainly dependent on the constraints’ density. For both MAX effector proteins, we statistically challenged the topological landscapes obtained from experimental pressure data against the landscapes generated from randomized-scrambled data (see Materials and Methods and Supplementary Materials, Figure S10). The statistical analysis involved α value rejection criteria (confidence level > 95%) for selecting clusters and building their fractional contact maps. We used two minimum size cluster parameters that played on the selection stringency (high stringency with a minimum size of 10, or 5 for lower stringency) to analyze the total merged populations of 47,570 and 49,874 conformers for AVR-Pia and AVR-Pib, respectively.

#### 2.4.2. Contact Maps of Statistically Relevant Clusters

**Figure 8:**
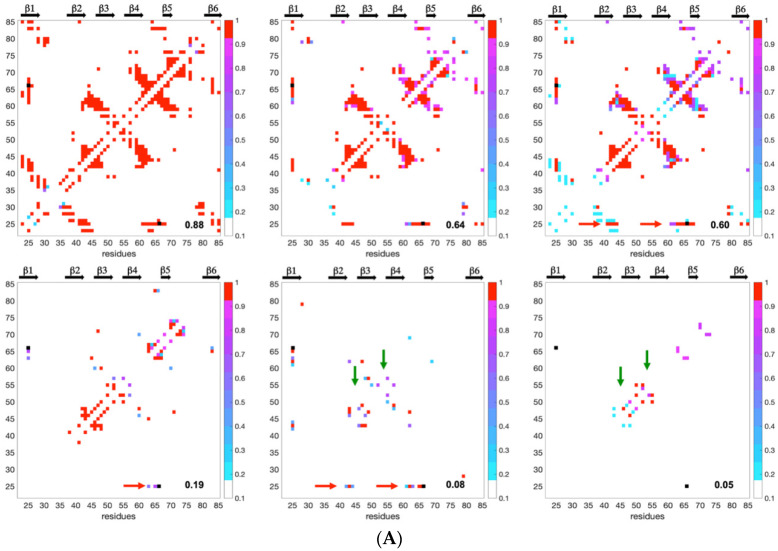

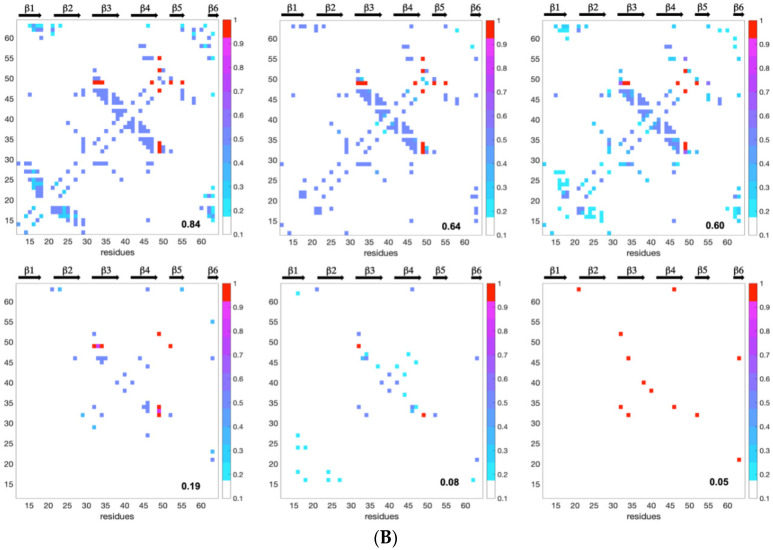
**Fractional contact map evolution upon pressure unfolding reaction** (**A**) for AVR-Pia and (**B**) for AVR-Pib. The Q values are given on the lower right corner of each contact map. The probability to find a contact is shown by a dot colored from red (probability of 1) to light blue colors (minimum probability of 0.2). The black dot for AVR-Pia corresponds to the C25-C66 di-SS bond. Black arrows at the top of each panel show strand secondary structures. Horizontal red arrows highlight contacts involving C25, and green vertical arrows indicate contacts in inter-strands turn regions found in AVR-Pia.

The fractional contact maps of conformers in statistically selected clusters (confidence level greater than 95%) were used to build a statistical view of the progress of residue–residue contacts during unfolding. In these maps, any fractional contact corresponds to the normalized frequency (0 to 1) of observing that contact in any of the selected cluster(s) at a given Q. All of the frames (60 and 67, for AVR-Pia and AVR-Pib, respectively) built with the default clustering minimum size are given in the Supplementary Materials videos (Videos S3 and S4). In Figure 8, we selected six fractional contact maps for describing the unfolding evolution, arranged from high to low Q values with the more restrictive clustering (minimum clustering size of 10). The top-left panels (Q = 0.88, 0.84) show inter-strands’ contacts involving residues in the ß1ß2, ß3ß4, ß4ß5 and ß1ß6 ß-sheets arrangements, formed by both almost native structures. Upon unfolding, from Q = 0.64 to 0.60, ß1ß2 inter-strands fractional contacts are preserved in AVR-Pia, through contacts involving Cys25, while they have low probability in AVR-Pib (light blue colors). The contact maps at Q = 0.60 also suggest that the ß-sheet formed by strands ß3, ß4 and ß5 is present in AVR-Pia and AVR-Pib at this stage of the unfolding reaction. At Q = 0.19 the contact maps show fractional contacts between residues in ß3 and ß4 for both AVR-Pia and AVR-Pib, indicating stable folding intermediates, that we previously called ß3ß4 folding intermediates, having common features in both proteins.

When 0.05 ≤ Q ≤ 0.08, long-range contacts between ß3 and ß4 are still observable in AVR-Pib, while they disappear in AVR-Pia at Q = 0.05. The AVR-Pia Cys25-Cys66 disulfide bond is also intrinsically encoded in the contact maps through long-range contacts of neighboring residues to Cys25 that are observed at Q = 0.08. The disruption of the ß3ß4 folding intermediate occurs rather late for AVR-Pib, where long-range contacts between ß3 and ß4 strands are persistent at Q = 0.05. For AVR-Pia, medium range contacts are observed that are the fingerprint of the turns and loop regions (inter-strand regions) that unfold at the final stage of the reaction. On average, the intensity of fractional contacts is higher (red and dark purple colors) for AVR-Pia than for AVR-Pib (lighter purple and blue colors), indicating a higher contact variability for AVR-Pib. This was already noticed at the level of the conformer distributions when comparing the spreading of the landscapes (Figure 5), where the conformer diversity was greater in the case of AVR-Pib than for AVR-Pia.

## 3. Discussion

To determine experimentally how the folding pathways of a protein differ, how the sub-structures are assembled, was a long-standing challenge. Based on experimental high pressure NMR data, we aimed to resolve whether sub-structures’ formation during folding (or melting during unfolding) could be disentangled from a complex conformational landscape.

High-pressure NMR analysis allowed the determination of residue specific native state probabilities along the range of pressure from 1 to 2500 bars. In this approach, the native state of a residue *i* is defined by (i) the inter-atomic distances measured in the X-ray structures between Cα*^i^* and the Cα of all other residues having their Cα atom within a sphere of 9 Å radius centered at Cα*^i^* and (ii) the φ*_i_*, ψ*_i_* values measured in the X-ray structures. The pressure-dependent residue specific native-state probabilities were translated into inter-residue contact probabilities that were filtered and used to restrain the conformer calculations by *Cyana3*. Then, the ensemble of conformers obtained from these calculations was used to build a conformational landscape. The conformers were clustered as a function of the reaction coordinate, Q, as order parameter. This is motivated by the fact that, in a funnel-like energy landscape, the energy of the conformations is reasonably correlated to the degree of *nativeness* [33]. We also controlled that the geometrical space sampling was strongly correlated with the energetics of the system, by calculating the free energy of the conformers.

**Figure 9:**
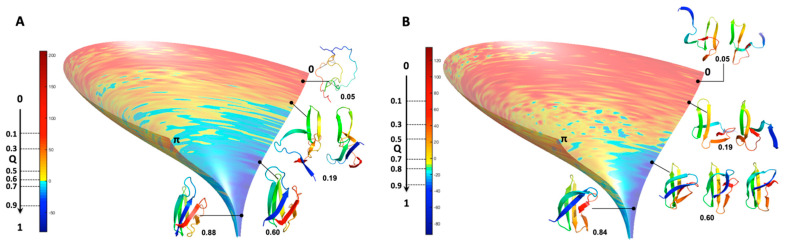
**Folding funnels computed for AVR-Pia and AVR-Pib.** Two identical idealized funnel-like surfaces were computed and the EvoEF2 energy values were projected on these surfaces for (**A**) AVR-Pia and (**B**) AVR-Pib. The equivalence between the EvoEF2 energy (given by the color ramp from blue to red) and the Q axis (from 1 to 0) was taken from the MP clusters average energies (Figure 6C). Cartoon representations at the given Q values are shown for selected intermediate conformers corresponding to most of the fractional contact maps given in Figure 8. For AVR-Pia at Q = 0.05 Cα-Cα contacts are shown by black doted lines in a mostly unfolded structure.

Since the distribution and density of the constraints along the protein sequence were different for the two MAX effectors, we introduced a statistical selection of conformer clusters retaining only the clusters that were statistically different from randomness. Accordingly, analyzing the landscapes was a targeted approach relying on (i) conformer clustering and (ii) statistical α-value rejection. This process allowed us to extract a discontinuous subset of clustered conformations and snapshots of probabilistic contact maps along Q. Through their probabilistic contact maps, Figure 8 describes the characteristics of inescapable pressure-induced conformers, some of which were used to illustrate Figure 9.

### 3.1. Folding Funnels

The agreement between the simulation results and the experimental data supports the idea that energetic frustration is indeed sufficiently reduced and that the protein folding mechanism, at least for small globular proteins, is strongly dependent on topological effects [33]. Thus, topological techniques were often found to be very powerful and were successfully applied to numerous problems in Physics, from theories of fundamental interactions to models of condensed matter [34]. Topology plays an important role in protein folding [35,36] and dynamics [37], and, in particular, for self-entanglement [38], as shown in a case study concerning the folding and unfolding of the slip-knotted AFV3-109 protein [39]. A hybrid strategy using topological simplification and energy calculation was applied to the nucleosome-folding problem [40] by using biased molecular dynamics, K-means clustering and the finite temperature string method [41], that can be applied both in the original Cartesian space of the system or in a set of collective variables.

As an alternative to choosing a set of collective variables that lacks generality, and to summarize our results, we simply preferred illustrating the folding funnels for both MAX effectors AVR-Pia and AVR-Pib. We computed an idealized half-funnel-like surface inside a 3D grid having a size of 100 regularly spaced values in the [0 π] interval by progressively constricting the surface with an arbitrary logarithmic function. The energy values shown in Figure 6B were resampled in the interval of minimum to maximum spreading <r.m.s.d.> at each Q value by applying a discrete cosine function algorithm [42] to expand and compress the energy matrix to a fixed size (100 × 100). This energy matrix was projected on the surface and used to color the half-funnel cartoon of AVR-Pia and AVR-Pib (Figure 9). The equivalence between Q axis and energy was taken from the “main populations” (MP) clusters’ average energies (Figure 6C).

As a consequence of the statistical selection analysis of conformers, we only focused on small parts having high reliability among the whole topology landscape of folding/unfolding under high-pressure. The initial collapse at low Q = 0.05 appears different between the two MAX effectors (short/medium contacts for AVR-Pia and long-range contacts for AVR-Pib), but illustrates only the most probable conformations. In both cases, these early conformations converge to a similar sub-structure folding hub (Q = 0.19) having ß3ß4 anti-parallel strands, while the orientation and position of other strands are not clearly defined. For AVR-Pia, the conformers at Q = 0.6 have an overall topology remarkably similar to the one shown at Q = 0.88. This is far from the case for AVR-Pib, where at Q = 0.6 different orientations are observed for the ß1 strand while ß2 adopts a better-defined orientation and position. In the last stage of the folding reaction (Q = 0.88 and Q = 0.84, for AVR-Pia and AVR-Pib, respectively) the conformers reach almost native topology.

### 3.2. Early Steps of Folding

Early long-range Cα–Cα contacts are established for AVR-Pib at low Q (0.05) and involve the residues G21, G32, S34, N38, R40, V46, G52 and V63. The limited number of allelic variants (eight variants) within the MAX effector AVR-Pib sub-family precludes drawing conclusions about the conservation patterns from the sequences. However, all of the residues previously listed are conserved, with the exception of V46 that is substituted by Ile in one of the sequences. The two hydrophobic residues, V46 and V63, are in contact, but most of the other interacting residues are glycine or charged/polar residues, and their sequence neighbors, while not strictly conserved, are all also charged or polar with one exception: the flanking neighbor residues of N38 are systematically hydrophobic. This early appearing long-range cohesion does not seem to be uniquely driven by a hydrophobic collapse and highlights the role of transient contacts between polar/charged residues in the early steps of the folding reaction of AVR-Pib. A similar behavior was previously reported for SH3 proteins; unfolding simulations and contact analysis demonstrated that differences in both hydrophobic interactions and side-chain hydrogen bonding interactions drive the folding/unfolding process [43].

The number of sequence variations is even smaller (five variants) in the case of the AVR-Pia sub-family. Intriguingly, the topological consequences of the conserved disulfide bond C25-C66 were rapidly observed during the folding reaction (Q = 0.08) with a set of Cα–Cα contacts between C25 and residues in the loop between ß4 and ß5. This suggests that the disulfide bond represents a major driving restraint that biases the folding landscape very early, restricting the overall divergence of conformers and simplifying (forcing) convergence to the native topology. Of course, since high-hydrostatic pressure is unable to break this covalent link, the likelihood of the results presented for AVR-Pia in this study depends, at least partially, on the fact that the disulfide bond formation constitutes the very first step of the protein folding, which might be not the case in vivo. It was shown by many experimental and theoretical studies that the pre-formed disulfide bonds can significantly increase the protein stability [44]. This underlines also some limitations of high-hydrostatic perturbation (as well as many other methods) for the study of protein folding, especially when highly energetic contacts such as covalent bonds are involved. More generally, there should be additional intermediates that were not captured because our analysis of HP-NMR unfolding is only sensitive to structural transitions that bias the topological landscape.

The impact of the guanidine concentration on the folding landscape should not be neglected. Very different sub-denaturant concentrations of guanidinium chloride were used to destabilize the 3D structures of AVR-Pia and AVR-Pib (4.5 M and 1.5 M, respectively) in order to trigger their unfolding reaction in the pressure range allowed by the experimental set-up (1–2500 bar). In a previous study [29], we showed that guanidine has a limited impact on ∆*V*, suggesting that the sub-denaturant concentrations of this harsh denaturant are able to slightly “smooth” the energy landscape by removing high-energy intermediates, while the main low-energy folding intermediates are maintained. The main effect of guanidine was found on the Transition State Ensemble (TSE) populated during protein unfolding. Indeed, P-jump kinetic experiments revealed that, for the same protein, a different sub-denaturing concentration of guanidine yields quantitative (more or less hydrated TSE) and qualitative (regions concerned) differences at the TSE level [29]. Thus, beside the important difference in their primary structure, the different sub-denaturant concentrations of guanidine used for AVR-Pia and AVR-Pib might impact, to some extent, the folding routes followed by these two proteins during the folding/unfolding reaction.

## 4. Materials and Methods

### 4.1. Protein Expression and Purification

The coding genes for AVR-Pia and AVR-Pib proteins were subcloned in pepL, a vector that allows the expression of a periplasm secretion signal peptide, and of a 6xHis-3C fusion protein. Constructs were then transformed into *E. coli* BL21(DE3) (Stratagene, Amsterdam, The Netherlands).

Uniform ^15^N labeling was obtained by growing cells in minimal M9 medium containing ^15^NH_4_Cl as the sole source of nitrogen. Protein was expressed overnight at 20 °C after induction with 0.2 mM IPTG. Cells were collected by centrifugation and suspended in 120 mL of cold lysis buffer comprising 200 mM Tris-HCl buffered at pH 8 and containing 500 mM sucrose, to which were added 40 mL of 5 mM EDTA buffered at pH 8, 40 mL of 0.1 mg/mL lysozyme, and 200 mL of TE buffer (200 mM Tris-HCl pH 8 and 0.5 mM EDTA). After 30 min of incubation on ice, 4 mL of MgSO_4_ were added and cell debris and insoluble materials were removed by centrifugation at 8000 rpm, for 30 min at 6 °C. The supernatant was loaded through a benchtop peristaltic pump onto a COmplete™ His-Tag Purification Column (Roche, Basel, Switzerland) equilibrated with buffer A (50 mM Tris-HCl pH 8, 300 mM NaCl, 0.1 mM benzamidine (and 1 mM DTT for AVR-Pia)). After elution with buffer B (buffer A supplemented with 500 mM imidazole), fractions containing the protein were dialyzed with homemade recombinant His-tagged 3C protease (mixed at 100:1 ratio) overnight at 4 °C in 10 mM Tris-HCl buffered at pH 7 for AVR-Pib and pH 8 for AVR-Pia, 150 mM NaCl (and 1 mM DTT for AVR-Pia). Cleavage was checked with SDS-PAGE and proteins were finally injected through an AKTA system into a Superdex S75 26/60 (GE Healthcare, Buc, France) column, equilibrated with 20 mM Tris-HCl buffered at pH 7 for AVR-Pib and pH 8 for AVR-Pia, 150 mM NaCl. The fractions containing the pure protein were pooled, concentrated to about 1 mM (protein concentration) and dialyzed overnight in 20 mM acetate pH 5.4, 100 mM NaCl for NMR experiments. Samples were then flash-frozen in liquid N2 and stored at −80 °C until NMR analysis.

### 4.2. Protein Unfolding

The 2D [^1^H,^15^N] HSQC were recorded on a Bruker AVANCE III 600 MHz spectrometer at 20 °C and at 15 different hydrostatic pressures (1, 50, 100, 300, 500, 700, 900, 1100, 1300, 1500, 1700, 1900, 2100, 2300 and 2500 bar). Samples with about 1 mM concentration of ^15^N-labeled proteins were used on 5 mm o.d. ceramic tubes (330 μL of sample volume) from Daedelus Innovations (Aston, PA, USA). Sub-denaturant concentration of guanidinium chloride (4.5 M and 1.5 M, for AVR-Pia and AVR-Pib, respectively) were added in order to trigger the protein stability into the pressure range allowed by the experimental set-up (1–2500 bar). Hydrostatic pressure was applied to the sample directly within the magnet using the Xtreme Syringe Pump (Daedelus Innovations). Each pressure jump was separated by a 2 h relaxation time, to allow the denaturation reaction to reach full equilibrium. Relaxation times for the folding/unfolding reactions were previously estimated from a series of 1D NMR experiments recorded after 200 bar P-Jump, following the increase in the resonance band corresponding to the methyl groups in the unfolded state of the protein. Note that the 1D spectra recorded at 1 bar on each protein sample before and after the full pressurization process gave strictly the same results, demonstrating the perfect reversibility of the folding/unfolding reaction.

In the case of AVR-Pia, the resonance assignment was already deposited at the BMRB (n° 25460), but under physical and chemical conditions slightly different than those used in the present study (20 mM Citrate pH 5.4, 100 mM NaCl, 4.5 M GuHCl). Since the presence of guanidine in the sample used for the denaturation experiments yields significant shifts in the position of the [^1^H,^15^N] HSQC cross-peaks, titration experiments were used to re-assign the amide cross-peaks in the conditions of the HP-NMR experiments. For AVR-Pib, we used [^1^H,^15^N] NOESY-HSQC (mixing time: 150 ms) and [^1^H,^15^N] TOCSY-HSQC (isotropic mixing time: 60 ms) 3D experiments to assign the amide group resonances in the condition of the denaturation study (20 mM Acetate buffer pH 5.4, 100 mM NaCl, 1.5 M GuHCl), following the classical sequential assignment strategy. Then, the intensities of the amide cross-peaks were measured for the folded species at each pressure and fitted with a two-state model:

(1) $$I=\frac{I_{u}+I_{f}e^{-\left(\Delta G_{f}^{0}+p\Delta V_{f}^{0}\right)/RT}}{1+e^{-\left(\Delta G_{f}^{0}+p\Delta V_{f}^{0}\right)/RT}}$$

In this equation, *I* is the cross-peak intensity measured at a given pressure, and I_f_ and *I_u_* correspond to the cross-peak intensities in the folded state (1 bar) and in the unfolded state (2500 bar), respectively. $\Delta G_{f}^{0}$ stands for the residue specific apparent free energy of folding at atmospheric pressure, and $\Delta V_{f}^{0}$ corresponds to the residue specific apparent volume of folding for pressure denaturation.

### 4.3. Topological Space Analysis

Let n Cα atoms, and *d_i,j_* the distance between the *i* and *j* Cα atoms, where *i* = 1, 2,…, n and *j* = 1, 2,…, n.

The native constraint list *C_c_*, for any *d_c_* cut-off, is given by filtering:

(2) $$C_{c}\left(i,j\right)=\left\{\begin{array}{c} keep,\;\;d_{i,j}\leq d_{c}\\ reject,\;\;d_{i,j}>d_{c} \end{array}\right.$$

with $\left|i-j\right|>1$.

The filtered constraint list $C_{c}^{f}\;$ for a filter F for Cα atoms *i* and *j*, is given by:

(3) $$F:\;C_{c}^{f}\left(i,j\right)=\left\{\begin{array}{c} C_{c}\left(i,j\right),\;\;P\left(i,j\right)\geq f\\ reject,\;\;P\left(i,j\right)<f \end{array}\right.$$

$P\left(i,j\right)\;$ is the probability of a contact between Cα atoms *i* and *j*, given by the geometric mean [19] expressed as:

(4) $$P\left(i,j\right)=\;\sqrt{p_{i\;}p_{j}}$$

where $p_{i}$ and $p_{j}$ are the statistical translations of the fraction (short cut as “the folded state probability” in the following) that each of the two residues *i* and *j* is in the folded state at a given pressure.

**Figure 10:**
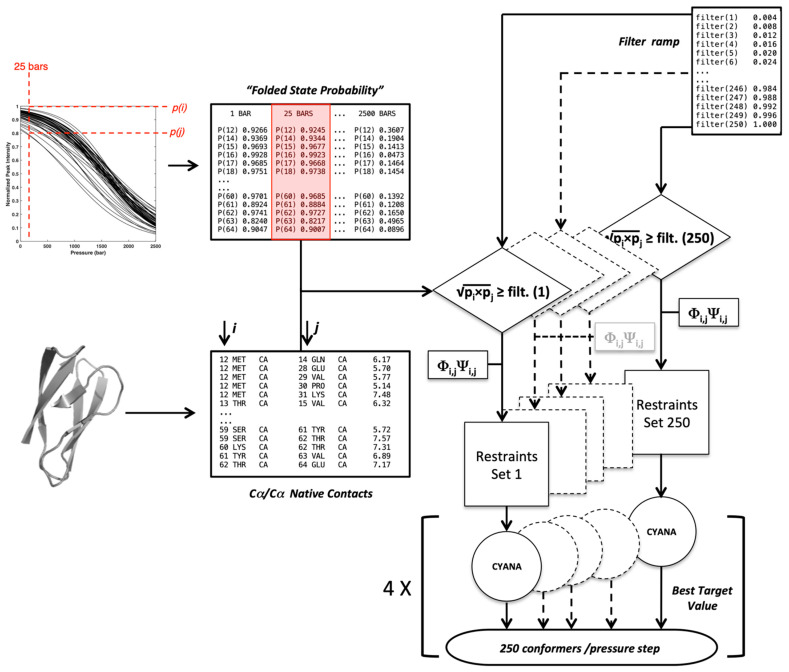
**Flowchart used to generate the conformers present in the proteins folding landscape.** The Cα–Cα contacts and the Φ/Ψ dihedral angles were measured from the native X-ray structures. For a given pressure, the folded state probabilities $p_{i}$ and $p_{j}$ were challenged against a filtering ramp to construct upper bound distance restraints’ files that were complemented by $\Phi_{i,j}\Psi_{i,j}$ dihedral restraints. Each of the 250 restraints sets was used as the input in *Cyana3*.

Let Q^T^ and Q^f^, the number of constraints in the $C_{c}$ and $C_{c}^{f}\;$ list, respectively, we define the fraction of native constraints for a filtered constraint list $C_{c}^{f}$ to be Q = Q^f^/Q^T^. The flowchart schematized below (Figure 10) gives the protocol used to generate a set of conformers at each pressure filtered by a selection F filter ramp. The population of conformers is then sorted by the fraction of native constraints per conformer (Q).

This flowchart crosses information coming from two main lists:

- A list of Cα–Cα distance upper bounds with a cutoff of 9 Å generated from the PDB structure 6Q76 (B chain), 5Z1V (A chain) for AVR-Pia and AVR-Pib, respectively. In addition, lists of backbone dihedral restraints (Φ/Ψ at ±10°) were also derived from the structures;
- A list containing the probability $p_{i}$ to find a residue *i* in a folded state, obtained from the normalized experimental residue-specific denaturation curve obtained for residue *i*. These curves are obtained from the fit of the intensity decrease with the pressure of HSQC cross-peak of residue *i* with Equation (1).

For a given pressure, 250 lists of contacts were established through filtering each native Cα–Cα contact by increasing cut-off values (f) obtained from a ramp (f = 0.004 to f = 1): a native contact between residues *i* and *j* is included in the list for a given f value if (according to Equation (4)):

$$\sqrt{p_{i}\cdot p_{j}}\geq f$$

Constraint lists ($C_{c}^{f}$) having zero or all of the native contacts were discarded. The Cα*_i_–*Cα*_j_* distance measured in the X-ray structures of the two MAX effectors was used as the upper bound limit to restrain the distance between residue *i* and *j* in the *Cyana3* calculations. In addition, the backbone Φ*_i_*/Ψ*_i_* and Φ*_j_*/Ψ*_j_* dihedral angles measured in the crystal structures were used as the constraints (±10°) only for the residues in the ß-strands to further restrain the available conformational space of the residues involved in contacts during calculations. This procedure was repeated at each pressure, from 1 to 2500 bar, with 25 bar steps.

### 4.4. Cyana Calculations

The torsion angle dynamics in *Cyana3*, restrained by the Cα–Cα upper bounds derived from each $C_{c}^{f}$ and backbone dihedral restraints, were used to generate one conformer (selected as having the best target function over 100 calculations) per pressure (from 1 to 2500 bar, with 25 bar steps) and per ramp cut-off (f). *Cyana3* implements the angle dynamics with minimization of a target function involving optimal geometry defined in a residue library that included Van der Waals atomic radii giving fine, well-defined Ramachandran conditions for the conformers. All calculations were repeated four times with different random conditions. All the models were sorted according to the fraction of native upper bound constraints (Q = Q^f^/Q^T^).

### 4.5. Energy Calculations

The internal energy of each conformer was calculated by evoEF2 software [32]. The conformer was first submitted with the *RepairStructure* option that optimizes the energy of the provided model by combinatorial exploration of the side-chain rotamers. Then, the resulting model was submitted to evoEF2 with the *ComputeStability* option to calculate the free energy (evoEF2 arbitrary units), and more specific contribution terms (Van der Waals and H-Bonds energies) of the model. The complete EvoEF2 energy function is written as:

E_EvoEF2_ = E_VDW_ + E_ELEC_ + E_HB_ + E_DESOLV_ + E_SS_ + E_AAPP_ + E_RAMA_ + E_ROT_ − E_REF_ (5)

Here, E_VDW_, E_ELEC_, E_HB_, E_DESOLV_ and E_REF_ represent the total Van der Waals, electrostatic, hydrogen bonding, de-solvation and reference energy terms for a protein system, respectively. The protein reference energy term, E_REF_, is used to model the energy of the protein in the unfolded state and it is calculated as the sum of amino acid-specific reference energy values [29]. In the four additional terms, E_SS_ describes the disulfide-bonding interactions, E_AAPP_ represents the energy for calculating amino acid propensities at given backbone (Φ/ψ) angles, E_RAMA_ is the Ramachandran term for choosing specific backbone angles (Φ /ψ) given a particular amino acid and E_ROT_ is the energy term for modeling the rotamer probabilities from the rotamer library. Difference in free energy expressed in kcal mol^−1^ is easily calculated from evoEF2 energy differences.

### 4.6. Clustering

We used the MaxCluster (http://www.sbg.bio.ic.ac.uk/~maxcluster/download.html, accessed on 15 May 2019) software that is fast enough for computing pairs of r.m.s.d. for a large set of structures (in the order of 104 structures). The Nearest Neighbor (NN) clustering algorithm in MaxCluster is based on the method of Shortle et al. [31]. The clustering must perform two goals: (i) global shape recognition and clustering at low Q values; and (ii) more local r.m.s.d. clustering at high Q values. Accordingly, we adapted MaxCluster parameters that perform well with these two tasks. Since we performed 4 *Cyana3* calculations for each constraint list, yielding four conformers per list, we choose the minimum number of conformers to form a cluster to be five (default value).

The ceiling threshold (T_m_) was let at the default value (8 Å) in order to be able to cluster conformers, according to their global shapes. The minimum threshold was set to 2.5 Å, with the possibility to regress by 1 Å, which has the effect of slightly increasing the number of clusters at high Q values and discriminating clusters having small local structure differences.

### 4.7. Contact Maps Statistics

Statistical tests assume a null hypothesis of no relationship or no difference between groups. Then, they determine whether the observed data fall outside of the range of values predicted by the null hypothesis.

The computational flowchart for generating randomly scrambled conformer populations (null hypothesis) is essentially identical to Figure 10, except that at each pressure and at each filter value (*f*) the residue specific data ($p_{i}$) were scrambled before generating the constraint files. That has the effect of de-correlating the pressure to sequence specificity, if any. In other words, it disconnects the pressure and structure. To perform the statistical analysis, we pooled these randomly scrambled conformer populations to the original ‘pressure’ data, and the obtained merged data at each Q were re-clustered with the default minimum cluster size or with minimum size of 10. Under such null hypothesis assumption, the statistical significance of any cluster was assessed by the alpha value threshold α < 0.05, which means that in a given cluster less than 5% of the conformers could be generated by random-scrambling. Accordingly, any cluster having more than 95% of conformers generated from the original ‘pressure’ dataset was selected to be statistically relevant. Fractional contact maps were computed from the upper bound restraint files used to generate these conformers. In these maps, any factional contact corresponds to the normalized frequency (0 to 1) of observing that contact in the selected cluster(s) of the original ‘pressure’ dataset.

Molecular structure Figures and Videos were executed with PyMOL v.1.6. [45]. The other Figures and Videos were executed with the MatLab 2019 package.

## 5. Conclusions

In this study, we demonstrate that the identification of folding intermediate conformations is feasible from a targeted geometrical sampling under control of HP-NMR data. The evolution of secondary structure formation or melting during folding/unfolding can be monitored at a residue level and furnishes insights into possible folding scenarios. In previous studies, it was already shown that a description of the conformational landscape of a protein at a given pressure was attainable by combining HP-NMR and molecular dynamic (Go-models) simulations [21,22]. Here, based on similar HP-NMR data, we succeeded in describing the full ensemble of conformers populated along the entire pressure axis, from 1 bar (native structure) to 2500 bar (unfolded structures). Among other things, this was made possible by replacing the Go-model simulations by faster *Cyana3* distance-geometry calculations. It should be stressed that, although our approach brings considerably more complete information, the calculation time needed remains reasonable and similar to that used in the previous method (2232 min. for the calculation of the full ensemble of conformers, with 10 CPU Intel Xeon E5-2660 @ 2.20 GHz).

Our strategy can be applied to other proteins of small/moderate sizes that are amenable to HP-NMR measurements. We believe that by identifying sub-structure hubs and intermediate conformations our approach will be valuable to drive meta-dynamics simulations upon pressure unfolding [46]. For example, it would be possible to investigate multiple pathways by combining individual contact maps’ information that would provide a better understanding of the energetics and kinetics of the pressure-induced unfolding of proteins.

## Data Availability

Not applicable.

## Supplementary Materials

The following are available online at https://www.mdpi.com/article/10.3390/ijms23105461/s1.

## Abbreviation

H(H)P-NMR: High (Hydrostatic) Pressure Nuclear Magnetic Resonance.
