# Supplementary material for: Combining High-Pressure NMR and Geometrical Sampling to Obtain a Full Topological Description of Protein Folding Landscapes: Application to the Folding of Two MAX Effectors from Magnaporthe oryzae
Source: Int J Mol Sci. 2022 May 13;23(10):5461. doi: 10.3390/ijms23105461 (PMC9141691; doi:10.3390/ijms23105461)
Supplement: Supplementary file 1 [file ijms-23-05461-s001.zip › ijms-1703794-supplementary 2.pdf]

Supplementary Materials

Table S1. Backbone r.m.s.d. at different cut-offs :

20 conformers were calculated for each protein with upper limit restraints filtered below different distance cut-offs, and with or without ϕ, ψ dihedral (± 10°) restraints. The r.m.s.d. (Å) of the backbone heavy atoms were calculated either between each pair of the 20 conformers (top values), or between the 20 conformers and the corresponding X-ray structure (bottom values).

	**Avr-Pia**		**Avr-Pib**	
	without ϕ, ψ	*with* ϕ, ψ	*without* ϕ, ψ	*with* ϕ, ψ
**Cut-off 7 Å**	4.79 ± 2.33<br>4.79 ± 1.83	2.60 ± 0.55<br>2.48 ± 0.62	6.65 ± 2.51<br>6.82 ± 2.72	5.92 ± 2.84<br>4.89 ± 2.80
**Cut-off 8 Å**	2.72 ± 0.43<br>2.77 ± 0.54	2.30 ± 0.58<br>2.06 ± 0.52	6.34 ± 2.29<br>6.59 ± 2.43	3.03 ± 0.76<br>2.82 ± 0.87
**Cut-off 9 Å**	2.62 ± 0.48<br>2.85 ± 0.62	2.26 ± 0.55<br>2.18 ± 0.64	5.83 ± 3.00<br>5.89 ± 3.01	1.78 ± 0.52<br>1.73 ± 0.34

***Figure S1**. **AVR-Pia (Top) and AVR-pib (Bottom) NMR fingerprints**, as indicated. [^{1}H-^{15}N] HSQC spectra were recorded at 800 MHz, 25°C on ≈ 1 mM ^{15}N-uniformly labeled samples dissolved in a 20 mM Sodium Acetate pH 5.4, 100 mM NaCl buffer and 4.5 M or 1.5 M guanidinium chloride for AVR-Pia and AVR-Pib, respectively. Cross-peak assignments are indicated using the one-letter amino acid and number code from the PDB.*

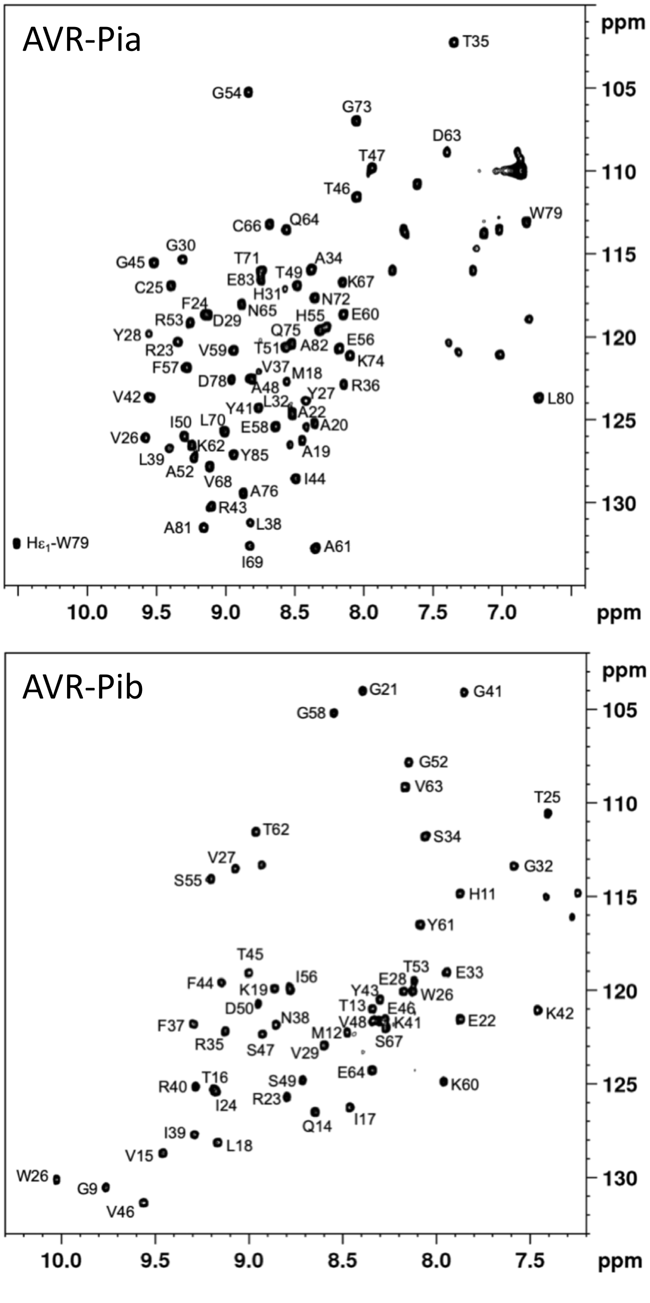

Figure S2. NMR detected high pressure unfolding of AVR-Pia *at 25°C and 4.5 M guanidinium chloride. (A-C) Examples of [^{1}H-^{15}N] HSQC NMR spectra at different pressures as indicated; (D) Overlay of three (residues T46, H55, and W79) residue-specific experimental denaturation curves obtained from the fits of the pressure-dependent sigmoidal decrease of the corresponding residue cross-peak intensities in the HSQC spectra with Eq. [1].*

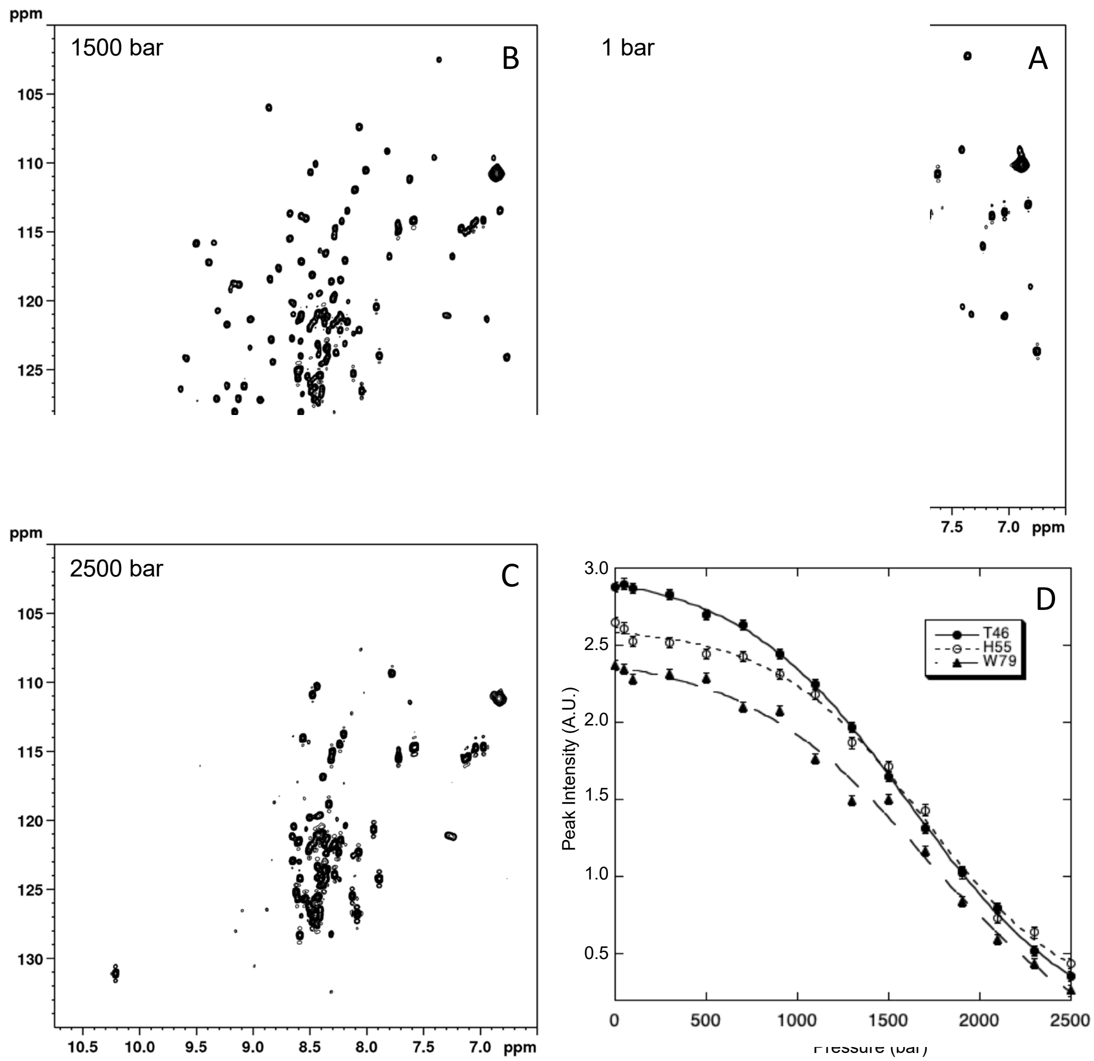

Figure S3. NMR detected high pressure unfolding of AVR-Pib *at 25°C and 1.5 M guanidinium chloride. (A-C) Examples of [^{1}H-^{15}N] HSQC NMR spectra at different pressures as indicated; (D) Overlay of three (residues V29, S47, and S55) residue-specific experimental denaturation curves obtained from the fits of the pressure-dependent sigmoidal decrease of the corresponding residue cross-peak intensities in the HSQC spectra with Eq. [1].*

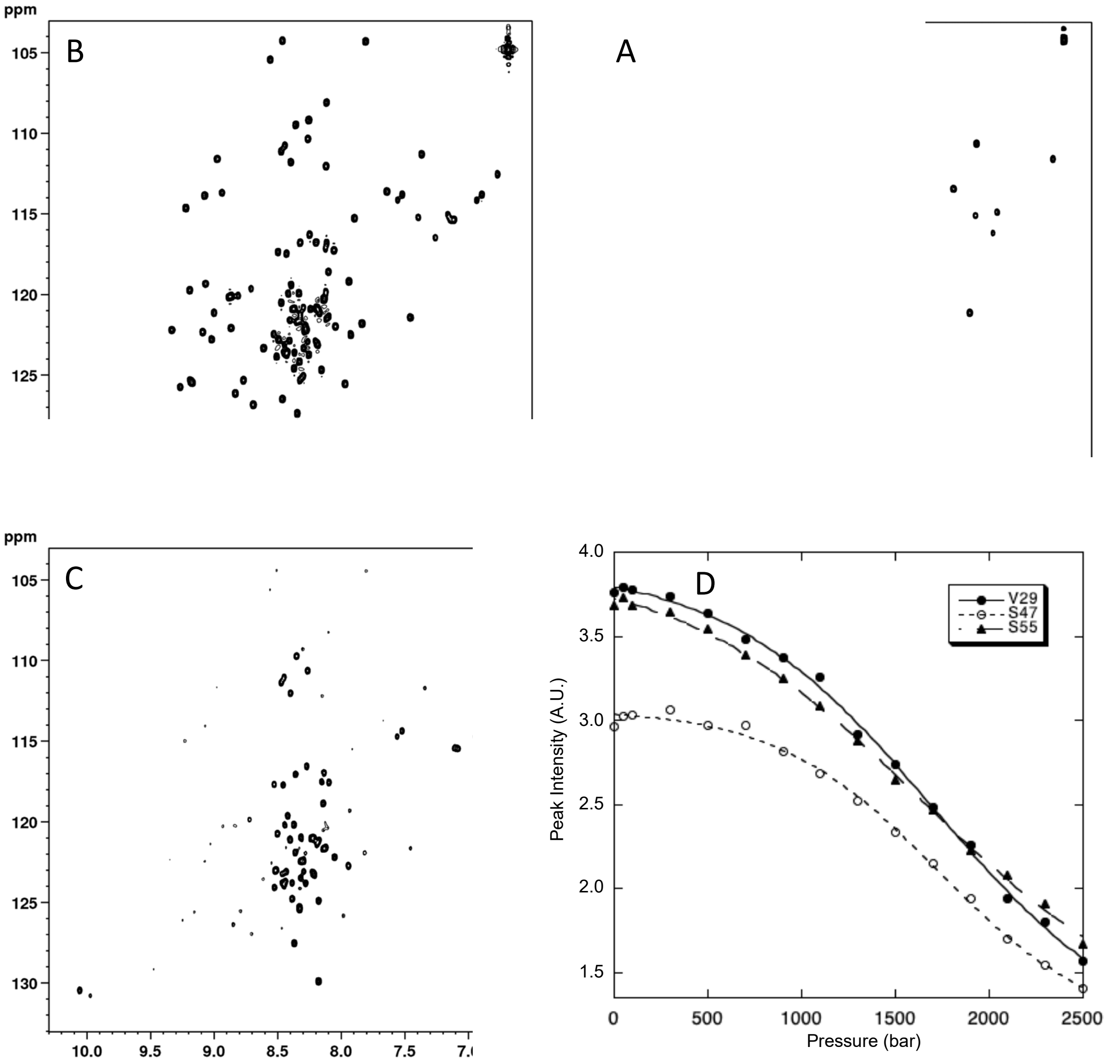

Figure S4. Steady-state thermodynamic parameters *obtained for (A) AVR-Pia and (B) AVR-Pib. Apparent residue-specific* ΔV_f^0 *(right pannels) and* ΔG_f^0 *(left pannels) values were obtained through the fit of the intensity decrease of the 2D [*^{1}*H-*^{15}*N] HSQC cross-peaks with pressure recorded on DEN4-ED3, and are plotted versus the protein sequence.*

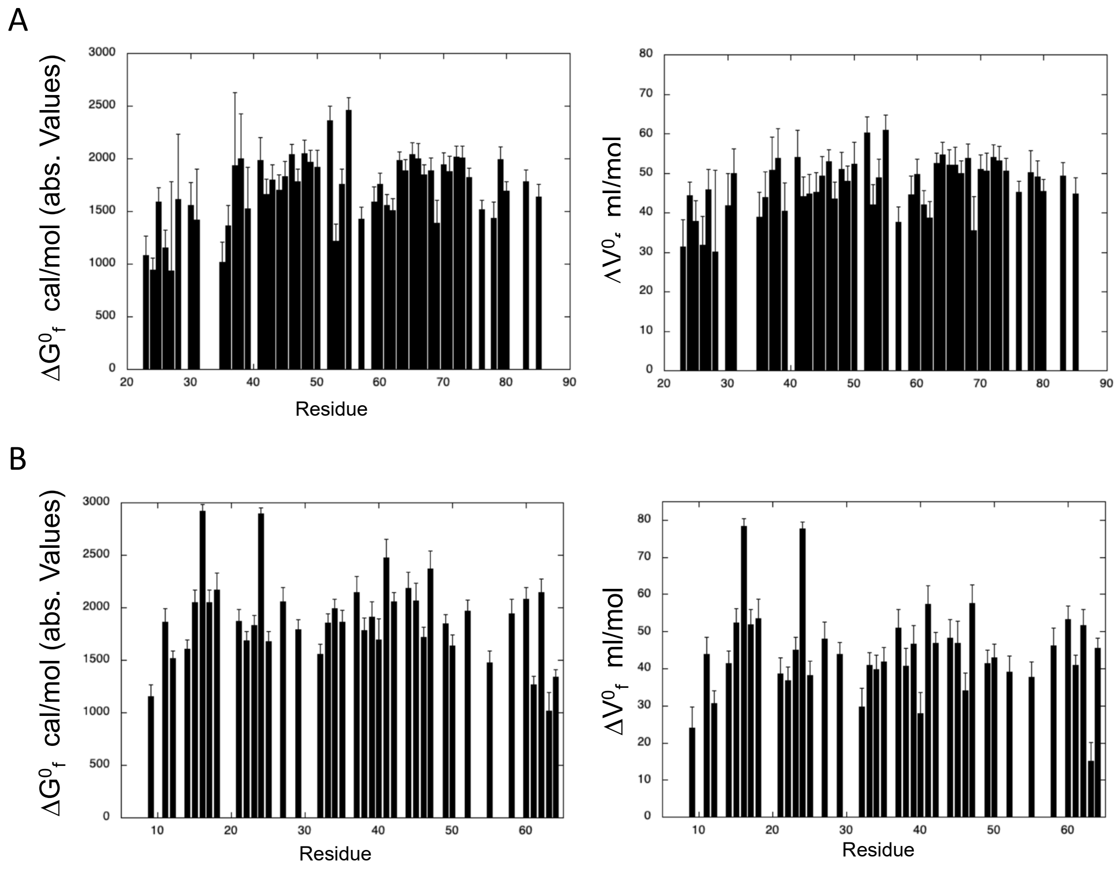

Figure S5. Unconstrained Conformational Sampling Using Cyana3

The Ramachandran landscape generated by Cyana3 was investigated by generating populations of 1000 conformers without any constraints for AVR-Pib and one C25-C66 disulphide bond for AVR-Pia. Validated structures with less or equal than (≤) 2 van der Waals violations were selected (684 and 694 conformers for AVR-Pia and AVR-Pib, respectively) and Ramachandran plots of all these selected conformers are overlaid below.

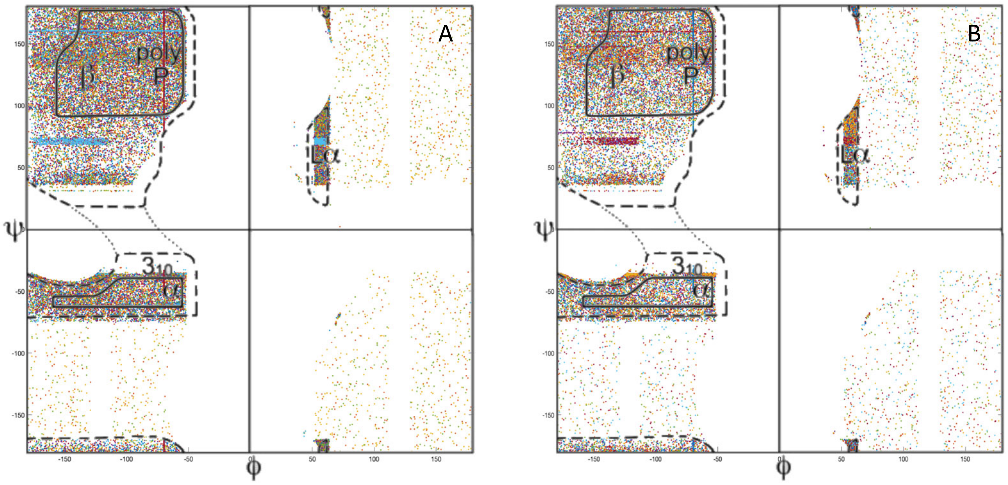

Ramachandran overlay plots of (A) AVR-Pia and (B) AVR-Pib.

A Ramachandran plot can be used to show in theory which values, or conformations, of the ψ and φ angles are possible for amino-acid residues in a protein. The ω angle at the peptide bond is 180° in *Cyana3* keeping the peptide in the trans-planar conformation. In the panels of the figure are shown the allowed φ,ψ backbone conformational regions from Ramachandran et al. 1963 and 1968 hard-sphere calculations: full radius in solid outline, reduced radius in dashed, and relaxed tau (N-Cα-C) angle in dotted lines (the γ-turns region is not indicated).

Ramachandran, G.N, Ramakrishnan, C., Sasisekharan, V. Stereochemistry of polypeptide chain configurations. *J Mol Biol.* **1963**, 7, 95-99.

Ramachandran, G.N., Sasisekharan, V. Conformation of polypeptides and proteins. *Adv Protein Chem.* **1968**, *23*, 283-438.

Figure S6. Determining the cut-off threshold for the constraints used for Cyana3 calculation of the structure of AVR-Pib*. Central panel: contact maps built from the X-Ray structure of AVR-Pib with cut-off threshold for the Cα-Cα distances of 7, 8 and 9 Å (from left to right), as indicated. Contacts below the diagonal correspond to residue where the distance to the corresponding Cα is lower than the threshold (black filled-squares). Above the diagonal, only the contacts for which fractional probability can be obtained have been reported (red filled-squares). In the upper panel, the corresponding Cα-Cα distances (70, 89 and 115 distances for cut-off thresholds of 7, 8 and 9 Å, respectively) have been reported on the X-ray structure. Residues involved in these contacts are also colored in red. The lower panel presents the results of the Cyana3 calculations using the corresponding Cα-Cα distances as upper bound limit restraints. The obtained models (the closest to the X-ray structure) (red cartoons) are superimposed with the X-ray structure of AVR-Pib (grey cartoons): <rmsd> of 6.82, 6.59 and 5.89 Å have been measured between the X-ray structures and the models obtained with cut-off threshold of 7, 8 and 9 Å, respectively (from left to right).*

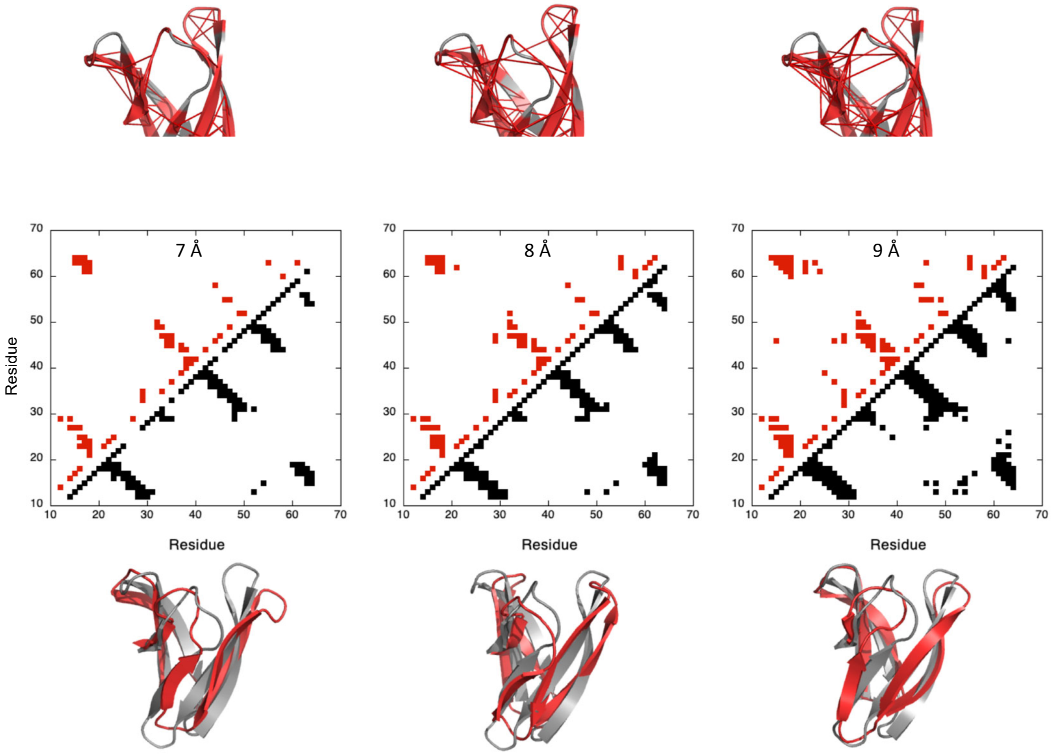

Figure S7. Superimposition (all Cα atoms) of conformers in clusters at Q=0.5

*(**A**) AVR-Pia single cluster and for AVR-Pib (**B**) shows the most populated cluster and (**C**) the second cluster (10 conformers). The average evoEF2 energy for the AVR-Pia cluster is +20.5 ± 6.1 a.u. The average evoEF2 energies for the AVR-Pib are +35.1 ± 4.6 and +42.6 ± 8.1 a.u., for the main cluster and the second cluster, respectively.*

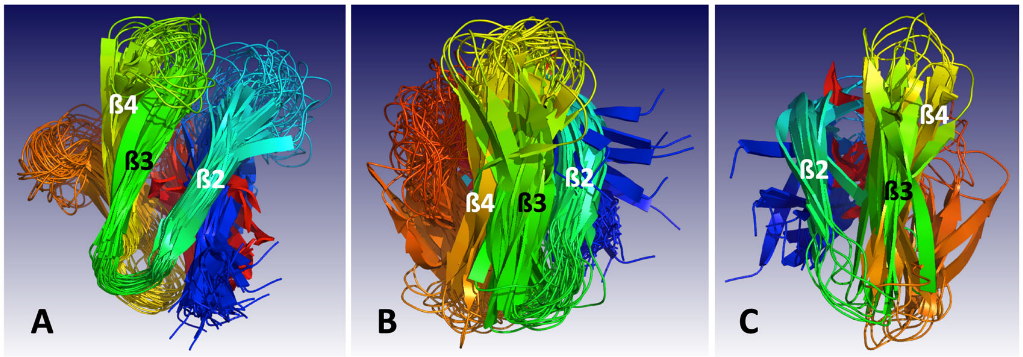

Figure S8. Evolution of van der Waals (VdW) and H-bonds energy terms for the conformer centroids of the most populated clusters.

The inter-residue VdW contributions are in red and green colors for AVR-Pia and AVR-Pib, respectively. The inter-residue H-bond contributions are in orange and blue colors for AVR-Pia and AVR-Pib, respectively. Calculated from evoEF2.

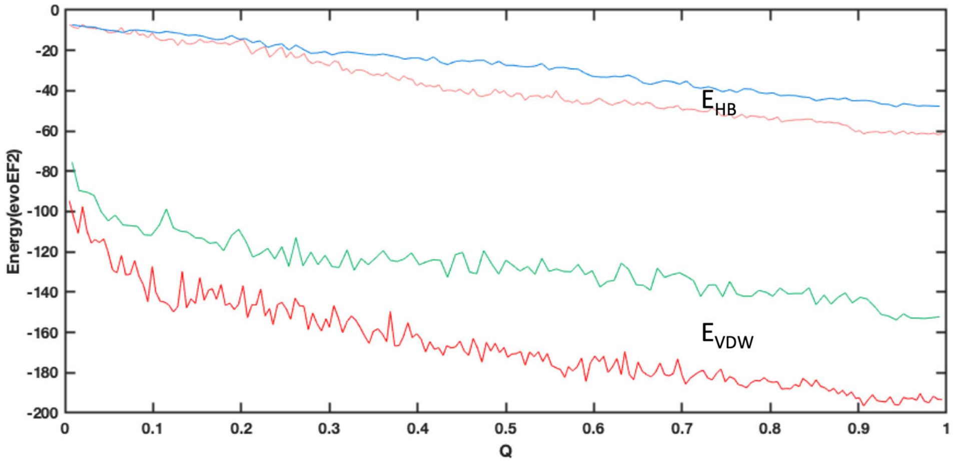

Figure S9. Cα-Cα distance restraints statistics *for (**A**) AVR-Pia and (**B**) AVR-Pib. The upper panels show the histograms of restraints distribution for the different ranges of |i-j|. The bottom panels are counts of medium range restraints (|i-j| < 5) in dark grey and long-range restraints in white. Only residues for which folded state probabilities could be measured were included. Calculated from Cyana3.*

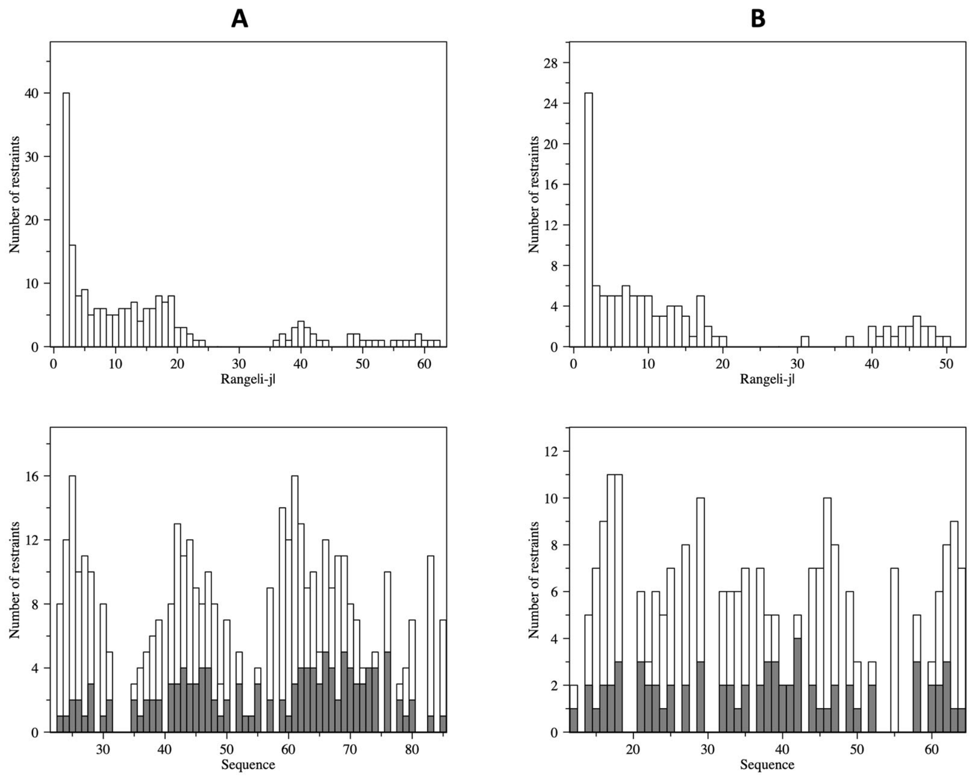

Figure S10. Superimposition of landscapes generated by randomly-scrambled data *(orange) for AVR-Pia (**A**) and AVR-Pib (**B**) on the corresponding High-Pressure-biased data (blue) reported of Figure 4A and 4B.*

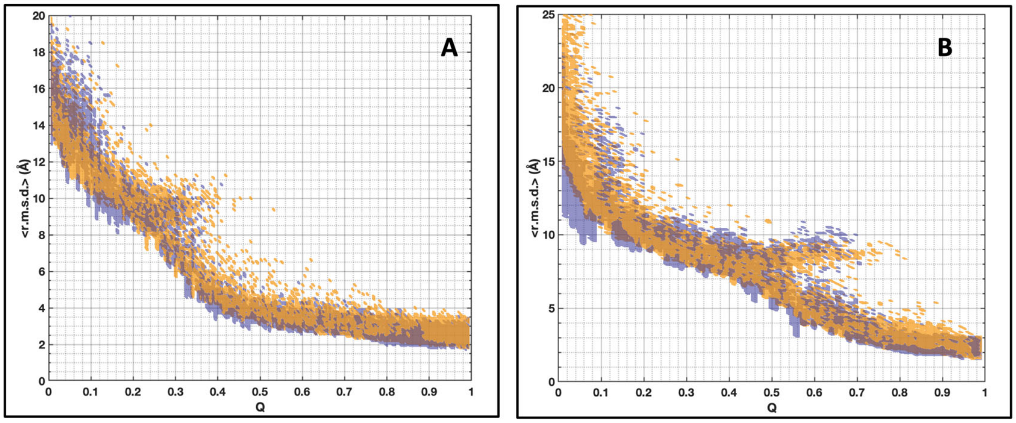

Video S1. Most populated clusters trajectory for AVR-Pia was build by extracting the conformer centroids of the most populated clusters at each Q value.

The cartoon representation from the N- to the C-terminus was rainbow coloured from dark blue to red and the ß-strands secondary structures found in the native structure are shown as cartoon arrows. Sticks show the disulfide bond between Cys25 and Cys66. The ß3 and ß4 strands are in green and yellow colours respectively. The frames were built by orienting by the ß3 - ß4 sheet in the vertical direction.

https://drive.google.com/file/d/1iQsXNhGYCnNe9sw52-MxKDkNk6t8FylH/view?usp=sharing

or available upon request to the authors.

Video S2. Most populated clusters trajectory for AVR-Pib was build by extracting the conformer centroids of the most populated clusters at each Q value.

The cartoon representation from the N- to the C-terminus was rainbow coloured from dark blue to red and the ß-strands secondary structures found in the native structure are shown as cartoon arrows. The ß3 and ß4 strands are in green and yellow colours respectively. The frames were built by orienting by the ß3 - ß4 sheet in the vertical direction.

https://drive.google.com/file/d/1O1JWWP7sGiiYFgg7VSlRz8C5I4miVuD5/view?usp=sharing

or available upon request to the authors.

Video S3. Fractional Contact Maps for AVR-Pia.

The first part of the video shows the evolution of fractional contact maps extracted (confidence level > 95%) from the merged landscape. The second part of the video shows the evolution of purely random (confidence level > 95%) fractional contact maps (extracted from the merged landscape). The minimum clustering size was set to 5.

https://drive.google.com/file/d/1t7FWXnVupOKECQ93RXNpkVmOX3paTyNt/view?usp=sharing

or available upon request to the authors.

Video S4. Fractional Contact Maps for AVR-Pib.
The first part of the video shows the evolution of fractional contact maps extracted (confidence level > 95%) from the merged landscape. The second part of the video shows the evolution of purely random (confidence level > 95%) fractional contact maps (extracted from the merged landscape). The minimum clustering size was set to 5.

https://drive.google.com/file/d/1EOD1eRrOzqyia6jg708H1r9lS1FXLCWc/view?usp=sharing

or available upon request to the authors.
